# Supplementary material for: Management of tuberculosis infection in Victorian children: A retrospective clinical audit of factors affecting treatment completion
Source: PLoS One. 2022 Oct 13;17(10):e0275789. doi: 10.1371/journal.pone.0275789 (PMC9562148; doi:10.1371/journal.pone.0275789)
Supplement: S1 Text — (DOCX) [file pone.0275789.s002.docx]

# S1 Text – Supplemental extended results document

**Management of tuberculosis infection in Victorian children: A retrospective clinical audit of factors affecting treatment completion**

#

**Contents**

[Table 1 – Extended patient characteristics 2](#_Toc111544177)

[Table 2 - Factors associated with TB preventive therapy completion showing model with all candidate variables and final model 4](#_Toc111544178)

[Table 3 - Type and clinical timing of TB screening tests 5](#_Toc111544179)

[Table 4 – Tuberculosis testing results from specimen processed at the Royal Children’s Hospital with test for concordance 6](#_Toc111544180)

[Table 5 - Clinical tests performed and results comparing those who completed therapy to those who did not complete 7](#_Toc111544181)

# Table 1 – Extended patient characteristics

**Patient characteristics of 402 children diagnosed with latent tuberculosis infection (TBI) at the Royal Children’s Hospital, Melbourne, 2010-2016, comparing Completed and Incomplete groups.**

| **Demographics** | **Total (%)** N=402 | **Completion Rate** | **Completed (%)** N=295 | **Incomplete (%)** N=107 | **P** |
| --- | --- | --- | --- | --- | --- |
| Infant (<1 yr) | 20 (5.0%) | 60% | 12(4.1%) | 8(7.5%) | 0.132 |
| Young Child (1-4 yrs) | 95(23.6%) | 82% | 77(26.1%) | 18 (16.8%) | - |
| Older Child (5-9 yrs) | 111 (27.6%) | 69% | 77(26.1%) | 34(31.8%) | - |
| Adolescent (10-17 yrs) | 176 (43.8%) | 73% | 129(43.7%) | 47 (43.9%) | - |
| **Sex** |  |  |  |  |  |
| Male | 213(53.0%) | 76% | 159(54.0%) | 54 (50.4%) | 0.542 |
| Female | 189 (47.0%) | 71% | 136(46.1%) | 53 (49.5%) | - |
| **Country of birth** |  |  |  |  |  |
| Australia | 91 (22.6%) | 65% | 58(19.7%) | 33(30.8%) | 0.018 |
| Other | 311 (77.4%) | 76% | 237(80.3%) | 74 (69.2%) | - |
| **Preferred language** |  |  |  |  |  |
| English | 146 (36.3%) | 66% | 94(31.9%) | 52 (48.6%) | <0.001 |
| Other | 249 (61.9%) | 80% | 199(67.5%) | 50 (46.7%) | - |
| Not recorded | 7 (1.7%) | 29% | 2 (0.7%) | 5 (4.7%) | - |
| **Highest risk factor** |  |  |  |  |  |
| Lived in endemic area | 155 (38.6%) | 79% | 122 (41.4%) | 33 (30.8 %) | 0.163 |
| Travelled in endemic area | 4 (1.0%) | 50% | 2 (0.7%) | 2 (1.9%) | - |
| Household contact with symptoms or TB | 165 (41.0%) | 68% | 112(37.8%) | 52(50.0%) | - |
| Non-household contact with symptoms or TB | 54 (13.4%) | 78% | 42(14.2%) | 12 (11.3%) | - |
| No documented exposure risks | 24 (6.0%) | 75% | 18(6.1%) | 6 (5.7%) | - |
| **Year of first visit** |  |  |  |  |  |
| 2010* | 30 (7.5%) | 72% | 21 (6.4%) | 9 (8.5%) | 0.001 |
| 2011 | 32 (8.0%) | 78% | 25 (8.5%) | 7 (6.6%) | - |
| 2012 | 46 (11.4%) | 80% | 37 (12.5%) | 9 (8.5%) | - |
| 2013 | 78 (19.4%) | 62% | 48 (16.2%) | 30 (28.3%) | - |
| 2014 | 78 (19.4%) | 73% | 57 (19.3%) | 21 (19.8%) | - |
| 2015 | 78 (19.4%) | 81% | 63 (21.3%) | 15 (14.2%) | - |
| 2016 | 55 (13.7%) | 82% | 45 (15.2%) | 10 (9.4%) | - |
| Not recorded | 5 (1.2%) | 0% | 0(0%) | 5 (4.7%) | - |
| **Referral source** |  |  |  |  |  |
| General Practitioner | 177 (44.03%) | 82.49 | 147 (49.83%) | 30 (28.04%) | 0.001 |
| Immigration health | 42 (10.45%) | 80.95 | 34 (11.49%) | 8 (7.55%) |  |
| Victorian TB Program | 139 (34.58%) | 61.87 | 87(28.81) | 54 (50.47%) |  |
| Other | 28 (6.97%) | 71.43 | 20 (6.76%) | 8 (7.55%) |  |
| Not recorded | 16 (3.98%) | 62.5 | 10 (3.39%) | 6 (5.61%) |  |
| **Clinic attended** |  |  |  |  |  |
| TB Clinic | 142 (35.32%) | 69.44 | 99 (33.56%) | 43 940.19%) | 0.062 |
| Infectious Disease | 42 (10.45%) | 76.32 | 34 (11.53%) | 8(7.48%) |  |
| Immigrant Health | 116 (28.86%) | 80.87 | 90 (30.51%) | 26 (24.3%) |  |
| Other General Medicine | 49 (12.19%) | 63.46 | 231 (10.51%) | 18 (16.82%) |  |
| Other | 32 (7.96%) | 87.88 | 28 (9.49%) | 4 (3.74%) |  |
| Not recorded | 21 (5.22%) | 60 | 13 (4.41%) | 8 (7.48%) |  |
| **Refugee health screening** |  |  |  |  |  |
| Yes | 182 (45.3%) | 85% | 154 (52.0%) | 28 (26.4%) | <0.001 |
| No | 220 (54.7%) | 65% | 142 (48.0%) | 78 (73.6%) | - |
| **Other conditions treated** |  |  |  |  |  |
| Yes | 230 (57.2%) | 81% | 186 (62.8%) | 44 (41.5%) | <0.001 |
| No | 172(42.8%) | 64% | 110 (37.2%) | 62 (58.5%) | - |
| **TPT regimen** |  |  |  |  |  |
| 6H | 377 (93.8%) | 75% | 283 (95.6%) | 95 (88.7%) | 0.001 |
| Other | 21 (5.2%) | 62% | 13(4.4%) | 8 (7.6%) | - |
| Not recorded | 4 (1%) | 0% | 0(0%) | 4 (3.8%) | - |
| **Medication changed** |  |  |  |  |  |
| Yes | 11(2.7%) | 73% | 8(2.7%) | 3 (2.8%) | 1 |
| No | 391 (97.3%) | 74% | 288 (97.3%) | 103 (97.2%) | - |
| **Side effects** |  |  |  |  |  |
| Yes | 15 (3.7%) | 47% | 7(2.4%) | 8 (7.6%) | 0.016 |
| No | 387 (96.3%) | 75% | 286 (97.6%) | 98 (92.5%) | - |

Notes: *3 participants were first seen prior to 2010 but had subsequent appointments between 2010-2016. P-values reflect test of difference in proportions comparing Complete and Incomplete groups using Pearson’s chi-squared test. 6H = six-month daily isoniazid; TB = tuberculosis; TPT = Tuberculosis preventive therapy.

# Table 2 - Factors associated with TB preventive therapy completion showing model with all candidate variables and final model

| **Variable** | **Model with all candidate variables** | | **Final model** | |
| --- | --- | --- | --- | --- |
|  | **OR** | **95% CI** | **OR** | **95% CI** |
| Age (years) | 0.99 | 0.93-1.05 | - | - |
| Male sex (vs female) | 1.31 | 0.76-2.25 | - | - |
| Overseas born (vs Australia) | 0.64 | 0.29-1.41 | - | - |
| Other language (vs English) | 1.34 | 0.72-2.51 | 1.45 | 0.88-2.39 |
| Referral source (vs GP)  - Department of Immigration  - Victorian TB program  - Detention health provider  - RCH internal | 2.01  0.79  0.10  0.73 | 0.50-8.13  0.27-2.30  0.02-0.49  0.7-7.37 | -  -  -  - | -  -  -  - |
| Clinic attended (vs TB clinic)  - Infectious disease clinic  - Immigrant health clinic  - Other | 0.37  0.34  0.34 | 0.11-1.27  0.12-0.98  0.02-5.03 | -  -  - | -  -  - |
| Level of risk (vs None)  - Travel in endemic area  - Lived in endemic area  - Non-household contact  - Household contact | 0.06  0.52  0.72  0.58 | 0.00-0.92  0.11-2.49  0.13-4.01  0.11-2.99 | -  -  -  - | -  -  -  - |
| Changed clinic | 0.60 | 0.23-1.56 | - | - |
| TPT regimen (vs 6H) | 1.42 | 0.22-9.12 | - | - |
| Target duration (months) | 1.10 | 0.52-2.35 | - | - |
| Medication changed | 2.89 | 0.54-15.51 | - | - |
| Refugee health screen | 2.55 | 1.04-6.27 | 2.31 | 1.34-4.00 |
| Other conditions treated | 1.78 | 0.93-3.41 | 1.67 | 1.0-2.85 |
| Medication side effects | 0.24 | 0.07-0.88 | 0.32 | 0.11- 0.94 |
| TB testing in Community | 1.11 | 0.46-2.68 | - | - |
| TB testing at RCH | 1.64 | 0.85-3.15 | - | - |

Notes: 6H = 6 month isoniazid regimen; CI = Confidence interval; GP = General Practitioner; OR = Odds ratio; RCH = Royal Children’s Hospital; TB = tuberculosis; TPT = Tuberculosis Preventive Therapy.

# Table 3 - Type and clinical timing of TB screening tests

| **Type of TB Testing** |  | **Total (%)** | **Completion Rate** |
| --- | --- | --- | --- |
| Community TST or IGRA | Yes | 282 (70.2%) | 72% |
|  | No | 115 (28.6%) | 78% |
|  | Not recorded | 5 (1.2%) | 80% |
| RCH TST or IGRA | Yes | 224 (55.7) | 79% |
|  | No | 178 (44.3%) | 67% |
| TST RESULT | TST Positive | 271 (67.4%) | 71% |
|  | TST Negative | 75 (18.7%) | 79% |
|  | Not recorded | 56 (13.9%) | 79% |
| IGRA RESULT | IGRA Positive | 111 (27.6%) | 74% |
|  | IGRA Negative | 89 (22.1%) | 76% |
|  | IGRA Indeterminate | 4(1%) | 75% |
|  | IGRA Not Performed | 198 (49.3%) | 72% |

Notes: TST – tuberculin skin est; IGRA = interferon gamma release assay; TST positivity based on risk-level according to national guidelines; IGRA result based on manufacturer assay reporting, where indeterminate indicates the result is not clearly positive or negative and hence likelihood of M.tuberculosis infection is uncertain.

# Table 4 – Tuberculosis testing results from specimen processed at the Royal Children’s Hospital with test for concordance

| RCH TST vs IGRA results | Total (%)  N=402 | Kappa  0.027 |
| --- | --- | --- |
| TST + (no IGRA) | 148 (36.8%) |  |
| IGRA + (no TST) | 44 (10.9%) |  |
| TST+/IGRA+ | 51 (12. 7%) |  |
| TST +/IGRA -* | 72 (17.9%) |  |
| TST-/IGRA+ | 16 (4%) |  |
| TST-/ IGRA-* | 19 (4.7%) |  |
| TST- only | 40 (10%) |  |
| IGRA- only | 4 (1%) |  |
| Neither test recorded | 10 (2.5%) |  |

Notes: TST – tuberculin skin est; IGRA = interferon gamma release assay; TST positivity based on risk-level according to national guidelines; IGRA result based on manufacturer assay reporting, where indeterminate indicates the result is not clearly positive or negative and hence likelihood of M.tuberculosis infection is uncertain. *Indeterminate results included with IGRA- for this comparison. Cohen’s kappa shows weak/no agreement between the TST and IGRA results, however it has limited use in this population as it is highly influenced by background prevalence.

# Table 5 - Clinical tests performed and results comparing those who completed therapy to those who did not complete

| **Type of Test** |  | **Total (%)** | **Completion Rate** | **Completed Therapy (%)** | **Not Complete (%)** | **P** |
| --- | --- | --- | --- | --- | --- | --- |
| CXR | Normal | 307 (76.37%) | 72.31 | 222 (75%) | 85 (80.19%) | 0.418 |
|  | Abnormal | 82 (20.4%) | 79.27 | 65 (21.96%) | 17 (16.04%) |  |
|  | Not recorded | 13 (3.23%) | 69.23 | 9 (3.04%) | 4 (3.77%) |  |
| LFTS | Normal | 213 (52.99%) | 77 | 164 (55.41%) | 49 (46.23) | 0.002 |
|  | Abnormal | 35 (8.71%) | 91.43 | 32 (10.81%) | 3 (2.83) |  |
|  | Not recorded | 154 (38.31%) | 64.94 | 100 (33.78%) | 54 (50.94%) |  |
| FBE/ESR/CRP | Yes | 265 (65.92%) | 78.49 | 208 (70.27%) | 57 (53.77%) | 0.002 |
|  | No | 137 (34.08%) | 64.23 | 88 (29.73%) | 49 (46.23%) |  |
| Other Tests | Yes | 288 (71.64%) | 80.21 | 231 (78.04%) | 57 (53.77%) | <0.001 |
|  | No | 114 (28.36%) | 57.02 | 65 (21.96%) | 49 (46.23%) |  |

P-values reflect test of difference in probability of completion between different groups using Pearson’s chi-squared test.
