# Supplementary material for: Management of tuberculosis infection in Victorian children: A retrospective clinical audit of factors affecting treatment completion
Source: PLoS One. 2022 Oct 13;17(10):e0275789. doi: 10.1371/journal.pone.0275789 (PMC9562148; doi:10.1371/journal.pone.0275789)
Supplement: Attachment — Submitted filename: Response to Reviewers.docx [file pone.0275789.s004.docx]

# **Response to Reviewers and Editor**

PONE-D-21-40361

Management of tuberculosis infection in Victorian children: A retrospective clinical audit of factors affecting treatment completion

Thank you for your Reviews and Editorial feedback, including request for Minor Revision. We have revised the manuscript according to your suggestions and providing the additional information requested – see details below.

Please find attached:

- A marked-up copy of our manuscript that highlights changes made to the original version.
- An unmarked version of your revised paper without tracked changes.
- Updated supplemental material document
- New supplemental data analysis file

Journal Requirements:

When submitting your revision, we need you to address these additional requirements.

1. Please ensure that your manuscript meets PLOS ONE's style requirements, including those for file naming. The PLOS ONE style templates can be found at 
https://journals.plos.org/plosone/s/file?id=wjVg/PLOSOne_formatting_sample_main_body.pdf and 
https://journals.plos.org/plosone/s/file?id=ba62/PLOSOne_formatting_sample_title_authors_affiliations.pdf

Checked and updated formatting.

2. Thank you for stating the following in the Competing Interests section: "HG, SG, ST, SK, SR are employed by the Royal Children’s Hospital and work in the TB clinic."

Please confirm that this does not alter your adherence to all PLOS ONE policies on sharing data and materials, by including the following statement: "This does not alter our adherence to PLOS ONE policies on sharing data and materials.” (as detailed online in our guide for authors http://journals.plos.org/plosone/s/competing-interests).  If there are restrictions on sharing of data and/or materials, please state these. Please note that we cannot proceed with consideration of your article until this information has been declared.

Please include your updated Competing Interests statement in your cover letter; we will change the online submission form on your behalf.

Updated and included in Cover Letter as requested.

3. In your Data Availability statement, you have not specified where the minimal data set underlying the results described in your manuscript can be found. PLOS defines a study's minimal data set as the underlying data used to reach the conclusions drawn in the manuscript and any additional data required to replicate the reported study findings in their entirety. All PLOS journals require that the minimal data set be made fully available. For more information about our data policy, please see http://journals.plos.org/plosone/s/data-availability.

Upon re-submitting your revised manuscript, please upload your study’s minimal underlying data set as either Supporting Information files or to a stable, public repository and include the relevant URLs, DOIs, or accession numbers within your revised cover letter. For a list of acceptable repositories, please see http://journals.plos.org/plosone/s/data-availability#loc-recommended-repositories. Any potentially identifying patient information must be fully anonymized.

Important: If there are ethical or legal restrictions to sharing your data publicly, please explain these restrictions in detail. Please see our guidelines for more information on what we consider unacceptable restrictions to publicly sharing data: http://journals.plos.org/plosone/s/data-availability#loc-unacceptable-data-access-restrictions. Note that it is not acceptable for the authors to be the sole named individuals responsible for ensuring data access.

We will update your Data Availability statement to reflect the information you provide in your cover letter.

Our audit was conducted under the Royal Children's Hospital permissions for quality improvement activities and does not include permission to share individual patient data in an open access repository. We can share minimum data through data sharing agreements with individuals/institutions on request. We can also make the complete analysis file openly available for transparency on what we have done.

Updated information on data sharing: Deidentified data summaries and analysis file are provided in supplemental material. Our audit was conducted under the Royal Children's Hospital permissions for quality improvement activities and does not include permission to share individual patient data in an open access repository. The data underlying the results presented in the study are available for researchers who meet the criteria for access to confidential data from The Royal Children's Hospital HREC ([rch.ethics@rch.org.au](mailto:rch.ethics@rch.org.au); +61 393455044).

4. Your ethics statement should only appear in the Methods section of your manuscript. If your ethics statement is written in any section besides the Methods, please move it to the Methods section and delete it from any other section. Please ensure that your ethics statement is included in your manuscript, as the ethics statement entered into the online submission form will not be published alongside your manuscript.

We have removed the ethics statement from additional information section and retained it in the Methods.

5. Please include captions for your Supporting Information files at the end of your manuscript, and update any in-text citations to match accordingly. Please see our Supporting Information guidelines for more information: <http://journals.plos.org/plosone/s/supporting-information>.

Formatting revised accordingly.

6. Please review your reference list to ensure that it is complete and correct. If you have cited papers that have been retracted, please include the rationale for doing so in the manuscript text, or remove these references and replace them with relevant current references. Any changes to the reference list should be mentioned in the rebuttal letter that accompanies your revised manuscript. If you need to cite a retracted article, indicate the article’s retracted status in the References list and also include a citation and full reference for the retraction notice.

Checked.


Reviewers' comments:

Reviewer's Responses to Questions

**Comments to the Author**

1. Is the manuscript technically sound, and do the data support the conclusions?

The manuscript must describe a technically sound piece of scientific research with data that supports the conclusions. Experiments must have been conducted rigorously, with appropriate controls, replication, and sample sizes. The conclusions must be drawn appropriately based on the data presented.

Reviewer #1: Yes

Reviewer #2: Yes

2. Has the statistical analysis been performed appropriately and rigorously?

Reviewer #1: Yes

Reviewer #2: Yes

3. Have the authors made all data underlying the findings in their manuscript fully available?

The [PLOS Data policy](http://www.plosone.org/static/policies.action#sharing) requires authors to make all data underlying the findings described in their manuscript fully available without restriction, with rare exception (please refer to the Data Availability Statement in the manuscript PDF file). The data should be provided as part of the manuscript or its supporting information, or deposited to a public repository. For example, in addition to summary statistics, the data points behind means, medians and variance measures should be available. If there are restrictions on publicly sharing data—e.g. participant privacy or use of data from a third party—those must be specified.

Reviewer #1: No

Reviewer #2: No

4. Is the manuscript presented in an intelligible fashion and written in standard English?

PLOS ONE does not copyedit accepted manuscripts, so the language in submitted articles must be clear, correct, and unambiguous. Any typographical or grammatical errors should be corrected at revision, so please note any specific errors here.

Reviewer #1: Yes

Reviewer #2: Yes

5. Review Comments to the Author

Please use the space provided to explain your answers to the questions above. You may also include additional comments for the author, including concerns about dual publication, research ethics, or publication ethics. (Please upload your review as an attachment if it exceeds 20,000 characters)

Reviewer #1: This is a well written and straight forward piece of research. The research team have examined completion of TB treatment in young people. It has been performed as a clinical quality improvement project for their service but I believe it has greater applicability to other services in higher income countries.

Thank you for your kind comments and your helpful suggestions below.

My queries relate to the classification of complete and incomplete groups. Is there potential for misclassification in these groups. The complete classification is discussed in limitations but this should be further up forward in the manuscript. Does 5 out of 6 months indicate a good clinical outcome of treatment? Can the authors include additional justification of the underpinnings of this choice? Also, the incomplete group are there any children in this group who are incomplete but who followed all clinical advice and so would be considered complete? that is a medical complication that interfered with treatment or other factors that interfered and may cause misclassification? I think that this can all be handled with some additional explanation in the text.

Thank you. We have revised the Methods to be clearer about the TPT completion definition and risk of misclassification.

“The primary outcome was TPT completion, defined as completing pharmacological treatment to within 1 month of a target duration for the specified agent (for instance, at least 5 months of a 6-month isoniazid course), confirmed by the treating clinician. We determined this by identifying the recommended treatment and date of commencement, then checking all subsequent clinical encounters to find clinical documentation about anticipated and actual completion dates. If the treating clinician determined that adherence was inadequate (e.g. missed days/weeks) and recommended restarting or lengthening the duration of treatment we judged TPT completion against the revised date. If a participant refused TPT or ceased TPT early for any reason (including adverse effects) they were considered incomplete. We set the ‘within 1 month’ cut-off as a balance between misclassifying actual completes as incomplete (e.g. demonstrated adherence at 5.5 months and completed the final few weeks without further review) and misclassifying actual incompletes as complete (e.g. adherent at 3 months but did not fill repeat script to complete therapy). We consider this to be a conservative definition of treatment completion with the most likely source of misclassification coming from children who completed treatment but never returned for final clinical review (i.e. misclassified as ‘incomplete’). By definition, children with TBI are asymptomatic and there are no clinical tests that can confirm whether TPT has been successful.”

Also, Table 2 in the Supplemental section presents the multivariate analyses. I believe this would be better incorporated as part of the manuscript. The table can be abbreviated to fit in the manuscript, but I think this information is helpful for the reader to have available without searching the Supplemental material.

Thankyou for your suggestion. We have moved an abbreviated multivariate analysis Table into the main text and retained the full table (with all variables) in the supplemental material.

Ln104 in the manuscript mentions the previous 10 years but at Ln88 the manuscript mentions a period of 7 years between 2010 and 2016. Please check and correct if necessary

Thank you. Corrected to past 7 years.

Supplementary information in Table 2 footer states "Odds ration" and should be corrected to ratio.

Corrected.

The short hand of 6H is used in Table 1 and in the footer of that table. Also appears in Figure 1. However, this is not described in text. It would be useful for the reader to be introduced to this while reading about isoniazid treatment in the text.

Added 6H abbreviation to the text.

Reviewer #2: The authors should be commended for examining TB preventive therapy among children. They appropriately recognize the limitations of a retrospective audit but laudably describe the use of the results to improve patient care. This is implementation science and the manuscript could have benefited from using an implementation science lens, but this is not requisite. A few clarifications are needed however:
1. Line 72, Intro 2nd paragraph “in younger children with…” something is missing here

Thank you. Deleted ‘with’.

2. Line 80, Intro, last paragraph, “parental barriers” – what kind of parental factors? Were parental factors, in fact, assessed?

Changed to individual, social, and clinical factors for consistency with Discussion/Conclusion.

3. Line 83, Intro, last paragraph “more effective TPT” – compared to what?

Compared to currently. Revised to state “… some of these factors may be modifiable by service providers to deliver TPT more effectively”.

4. Line 141-142, results “6% had no known exposure risks” – why were they tested?

These children were typically refugee-background from countries not considered high risk for TB but screened for TB as part of routine recommended refugee health arrival screening. Or TB testing as part of work up before starting immunosuppressive therapy. Added this to text.

5. Line 152 or line 210, results, “discharged without review” – kindly clarify how this happened?

Treating clinicians differed in how frequently they would review children and at what stage they would discharge patients. We remark on these in particular because it is likely that the treating clinician was happy that they would complete treatment without further review.

6. Line 173, results typo: “that”

Corrected.

7. Line 296, discussion “conservative clinical definition” – on what is this clinical definition based?

Full description of the TPT completion definition added in Methods.

The primary outcome was TPT completion, defined as completing pharmacological treatment to within 1 month of a target duration for the specified agent (for instance, at least 5 months of a 6-month isoniazid course), confirmed by the treating clinician. We determined this by identifying the recommended treatment and date of commencement, then checking all subsequent clinical encounters to find clinical documentation about anticipated and actual completion dates. If the treating clinician determined that adherence was inadequate (e.g. missed days/weeks) and recommended restarting or lengthening the duration of treatment we judged TPT completion against the revised date. If a participant refused TPT or ceased TPT early for any reason (including adverse effects) they were considered incomplete. We set the ‘within 1 month’ cut-off as a balance between misclassifying actual completes as incomplete (e.g. demonstrated adherence at 5.5 months and completed the final few weeks without further review) and misclassifying actual incompletes as complete (e.g. adherent at 3 months but did not fill repeat script to complete therapy). We consider this to be a conservative definition of treatment completion with the most likely source of misclassification coming from children who completed treatment but never returned for final clinical review (i.e. misclassified as ‘incomplete’). By definition, children with TBI are asymptomatic and there are no clinical tests that can confirm whether TPT has been successful.

8. Line 308 – individual, social, clinical factors – but earlier in the text the authors state “Individual, parental, institutional” – kindly be consistent

Changed to individual, social, and clinical factors for consistency.
